# Supplementary material for: Persistent neuroimmune alterations in children who are HIV-exposed but uninfected at age 6–7 years: Associations with language development in a South African birth cohort
Source: bioRxiv. 2026 Mar 11:2026.03.03.709328. Preprint. [Version 1] doi: 10.64898/2026.03.03.709328 (PMC13060972; doi:10.64898/2026.03.03.709328)
Supplement: Supplement 1 [file media-1.pdf]

Supplementary materials

Persistent neuroimmune alterations in children who are HIV-exposed and uninfected at age 6–7 years: Associations with language development in a South African birth cohort

Cesc Bertran-Cobo[1,2*], Frances C Robertson[2,3,4], Simone Williams[2,5], Tusekile Sarah Kangwa[1,2], Jenna Annandale[1,2], Jessica Ringshaw[2,5], Layla Bradford[2,5], Nadia Hoffman[1,2], Heather J Zar[5,6], Dan J Stein[1,2,7‡], Kirsten A Donald[2,5‡], Petrus J W Naudé[1,2‡], Catherine J Wedderburn[2,5,8‡]

[1]Department of Psychiatry and Mental Health, University of Cape Town, Cape Town, South Africa

[2]Neuroscience Institute, University of Cape Town, Cape Town, South Africa

[3]Department of Human Biology, University of Cape Town, Cape Town, South Africa

[4]Cape Universities Body Imaging Centre (CUBIC), Cape Town, South Africa

[5]Department of Paediatrics and Child Health, Red Cross War Memorial Children's Hospital, University of Cape Town, Cape Town, South Africa

[6]SAMRC Unit on Child & Adolescent Health, Red Cross War Memorial Children's Hospital, University of Cape Town, Cape Town, South Africa

[7]SAMRC Unit on Risk and Resilience in Mental Disorders, University of Cape Town, Cape Town, South Africa

[8]Department of Clinical Research, London School of Hygiene & Tropical Medicine, London, United Kingdom

[‡]Shared last authorship

***Correspondence**: Cesc Bertran-Cobo (BRTFRA009@uct.ac.za)

Keywords: HIV exposure, cytokine, maternal immune activation, magnetic resonance spectroscopy, brain development, monocyte activation

Table of contents

Supplementary Tables 3

Supplementary Table 1. Cross-sectional group differences in serum marker levels 3

1.1. Maternal serum markers during pregnancy (2nd–3rd trimester) 3

1.2. Infant serum markers at 6 weeks of age 4

1.3. Child serum markers at age 2 years 5

1.4. Child serum markers at age 3 years 6

1.5. Child serum markers at age 5 years 7

Supplementary Table 2. Linear mixed-effects models 8

Supplementary Table 3. Group differences in parietal brain metabolite levels at 6–7 years 12

Supplementary Table 4. Associations between maternal/child serum marker levels at different timepoints and child neurometabolite ratios to creatine at 6–7 years 14

Supplementary Table 5. Adjusted linear models exploring associations between maternal/child serum marker levels at different timepoints and midline parietal grey matter neurometabolite absolute concentrations at 6–7 years 16

Supplementary Table 6. Sensitivity analyses 17

6.1. Maternal age at delivery 17

6.2. Maternal alcohol use during pregnancy 18

6.3. Maternal depression during pregnancy 19

6.4. Maternal smoking during pregnancy 20

6.5. Exclusive breastfeeding duration 21

Supplementary Table 7. Early Learning Outcome Measure scores at 6–7 years 22

Supplementary Figures 23

Supplementary Figure 1. Maternal serum marker levels during pregnancy 23

Supplementary Figure 2. Child neurometabolite absolute concentrations at 6–7 years of age 24

Mediation analyses 25

Model 1 25

Model 2 26

Model 3 27

Supplementary Tables

Supplementary Table 1. Cross-sectional group differences in serum marker levels

1.1. Maternal serum markers during pregnancy (2nd–3rd trimester)

Marker type	Marker	HIV- moms (n=133)	HIV+ moms (n=83)	P value Crude	Corrected
Pro-inflammatory cytokines (pg/mL)	**GM-CSF**	**49.01** (**61.59**)	**32.26** (**30.86**)	**0.001**	**0.007**
	IFNγ	9.69 (8.19)	10.48 (10.86)	0.68	0.77
	IL-1β	1.72 (1.37)	1.84 (1.71)	0.66	0.77
	IL-2	2.26 (2.47)	2.15 (2.57)	0.41	0.56
	IL-5	2.28 (1.73)	2.58 (2.55)	0.65	0.78
	IL-6	2.48 (2.6)	1.66 (2.5)	0.10	0.27
	IL-7	10.6 (6.91)	9.7 (8.21)	0.88	0.88
	IL-8	3.96 (4.96)	3.4 (3.76)	0.31	0.56
	TNFα	5.44 (2.84)	5.67 (4.28)	0.12	0.31
Anti-inflammatory cytokines (pg/mL)	IL-4	38.13 (44.31)	22.11 (42.86)	0.05	0.16
	IL-10	12.02 (15.78)	10.95 (12.11)	0.31	0.56
	IL-12p70	3.73 (3.35)	3.73 (3.07)	0.35	0.56
	**IL-13**	**6.04** (**7.1**)	**3.34** (**5.04**)	**0.0196**	0.07
Monocyte activation markers (ng/mL)	**CD14**	**1582.92** (**742.01**)	**1994.69** (**802.75**)	**0.0002**	**0.002**
	CD163	555.36 (388.43)	545.82 (360.69)	0.88	0.93
Neuroinflammatory markers (ng/mL)	**NGAL**	**169.15** (**145.61**)	**138.41** (**123.55**)	**0.004**	**0.018**
	**MMP-9**	**1226.05** (**1022.48**)	**793.61** (**910.7**)	**0.00003**	**0.0006**
	YKL-40	36.41 (30.8)	34.37 (44.16)	0.47	0.60

Group data is presented as raw median (IQR). Statistical tests were conducted on log-scaled data. Analyses were corrected for multiple comparisons using the Benjamini-Hochberg procedure.

1.2. Infant serum markers at 6 weeks of age

**Marker type**	**Marker**	**CHU** (n=100)	**CHEU** (n=56)	**P value** Crude	Corrected
Pro-inflammatory cytokines (pg/mL)	GM-CSF	14.41 (16.69)	11.2 (13.63)	0.09	0.34
	IFNγ	5.74 (5.64)	4.78 (9.57)	0.53	0.83
	**IL-1β**	**1.15 (1.3)**	**0.85 (1.21)**	**0.0231**	0.14
	IL-2	1.11 (1.43)	1.1 (1.39)	0.67	0.83
	IL-5	1.38 (1.32)	1.72 (1.31)	0.09	0.34
	IL-6	1.58 (3.05)	1.4 (2.41)	0.51	0.83
	IL-7	5.75 (5.07)	5.92 (5.68)	0.49	0.83
	IL-8	6.95 (6.8)	7.5 (9.38)	0.99	0.99
	TNFα	18.19 (9.74)	17.13 (16.73)	0.35	0.70
Anti-inflammatory cytokines (pg/mL)	IL-4	16.92 (32.81)	8.32 (25.12)	0.20	0.52
	IL-10	12.46 (15.63)	11.09 (12.34)	0.60	0.83
	IL-12p70	2.24 (3.01)	1.9 (2.37)	0.14	0.41
	IL-13	3.39 (5.34)	2.95 (4.94)	0.99	0.99
Monocyte activation markers (ng/mL)	**CD14**	**1521.49 (652.66)**	**1716.6 (627.05)**	**0.0109**	0.20
	CD163	603.29 (427.77)	634.12 (436.03)	0.67	0.86
Neuroinflammatory markers (ng/mL)	**NGAL**	**88.4 (40.28)**	**74.29 (41.35)**	**0.0102**	0.13
	MMP-9	364.84 (252.23)	342.55 (483.68)	0.74	0.83
	YKL-40	30.65 (18.12)	29.49 (12.14)	0.47	0.73

Group data is presented as raw median (IQR). Statistical tests were conducted on log-scaled data. Analyses were corrected for multiple comparisons using the Benjamini-Hochberg procedure.

1.3. Child serum markers at age 2 years

**Marker type**	**Marker**	**CHU** (n=107)	**CHEU** (n=60)	**P value** Crude	Corrected
Pro-inflammatory cytokines (pg/mL)	GM-CSF	119.97 (147.63)	91.96 (135.1)	0.14	0.25
	IFNγ	10.32 (7.11)	6.98 (8.51)	0.11	0.25
	**IL-1β**	**2.2** (**1.89**)	**1.61** (**1.51**)	**0.0049**	0.07
	**IL-2**	**3.06** (**2.21**)	**2.21** (**1.59**)	**0.0081**	0.07
	IL-5	3.04 (3.31)	3.1 (2.43)	0.48	0.57
	IL-6	3.42 (2.84)	2.54 (2.7)	0.53	0.64
	IL-7	10.6 (6.5)	8.28 (5.14)	0.15	0.25
	IL-8	9.74 (15.41)	8.9 (15.04)	0.29	0.38
	TNFα	13.02 (8.65)	12.28 (7.64)	0.29	0.42
Anti-inflammatory cytokines (pg/mL)	**IL-4**	**40.47** (**42.65**)	**27.6** (**30.69**)	**0.0180**	0.08
	IL-10	22.08 (18.51)	17.89 (11.4)	0.10	0.25
	**IL-12p70**	**5.02** (**3.23**)	**4.17 (2.5)**	**0.0137**	0.08
	IL-13	10.04 (13.43)	6.64 (11.56)	0.08	0.25
Monocyte activation markers (ng/mL)	CD14	2170.58 (872.73)	2096.79 (884.5)	0.83	0.83
	CD163	626.82 (318.06)	674.68 (519.74)	0.14	0.25
Neuroinflammatory markers (ng/mL)	NGAL	159.46 (168.04)	156.39 (119.54)	0.63	0.70
	MMP-9	801.98 (600.32)	775.28 (647.17)	0.83	0.83
	YKL-40	27.87 (20.15)	29.58 (30.97)	0.10	0.17

Group data is presented as raw median (IQR). Statistical tests were conducted on log-scaled data. Analyses were corrected for multiple comparisons using the Benjamini-Hochberg procedure.

1.4. Child serum markers at age 3 years

**Marker type**	**Marker**	**CHU** (n=88)	**CHEU** (n=53)	**P value** Crude	Corrected
Pro-inflammatory cytokines (pg/mL)	**GM-CSF**	**66.75** (**95.11**)	**48.74** (**70.62**)	**0.0211**	0.19
	IFNγ	18.94 (13.55)	20.8 (16.48)	0.24	0.50
	IL-1β	2.78 (1.99)	2.81 (1.67)	0.94	0.94
	IL-2	2.62 (1.46)	2.62 (2.1)	0.56	0.81
	IL-5	3.14 (3.47)	2.79 (2.42)	0.06	0.37
	IL-6	4.9 (11.33)	4.14 (6.84)	0.84	0.94
	IL-7	11.67 (5.94)	12.7 (7.2)	0.25	0.50
	IL-8	18.65 (39.82)	18.05 (23.7)	0.0	0.95
	TNFα	12.76 (6.08)	12.28 (7.62)	0.66	0.91
Anti-inflammatory cytokines (pg/mL)	IL-4	49.32 (106.84)	39.95 (60.22)	0.58	0.81
	IL-10	10.49 (7.69)	9.38 (5.22)	0.11	0.40
	IL-12p70	3.58 (2.31)	4.12 (2.88)	0.14	0.43
	IL-13	11.6 (24.9)	11.72 (19.1)	0.78	0.93
Monocyte activation markers (ng/mL)	**CD14**	**2255.06** (**990.69**)	**2538.94** (**922.53**)	**0.0135**	0.24
	CD163	661.71 (330.35)	589.32 (334.17)	0.13	0.43
Neuroinflammatory markers (ng/mL)	NGAL	115.62 (65.2)	107.16 (58.34)	0.21	0.53
	MMP-9	901.36 (864.1)	910.74 (1573.06)	0.33	0.67
	YKL-40	27.13 (20.31)	26.33 (24.76)	0.95	0.95

Group data is presented as raw median (IQR). Statistical tests were conducted on log-scaled data. Analyses were corrected for multiple comparisons using the Benjamini-Hochberg procedure.

1.5. Child serum markers at age 5 years

**Marker type**	**Marker**	**CHU** (n=101)	**CHEU** (n=60)	**P value** Crude	Corrected
Pro-inflammatory cytokines (pg/mL)	GM-CSF	76.78 (84.96)	53.37 (66.61)	0.07	0.27
	IFNγ	16.3 (9.7)	17.8 (8.26)	0.44	0.58
	IL-1β	2.77 (1.69)	2.4 (1.56)	0.43	0.61
	IL-2	2.42 (1.83)	2.12 (1.74)	0.20	0.50
	**IL-5**	**3.22** (**2.73**)	**2.3** (**2.21**)	**0.0102**	0.09
	IL-6	3.15 (7.48)	2.08 (4.27)	0.13	0.48
	IL-7	10.28 (7.5)	11.99 (8.4)	0.65	0.73
	IL-8	12.7 (32.83)	18.15 (42.38)	0.25	0.50
	TNFα	9.9 (5.34)	9.51 (8.65)	0.76	0.76
Anti-inflammatory cytokines (pg/mL)	IL-4	36.18 (109.09)	29.01 (41.65)	0.28	0.58
	**IL-10**	**9.13** (**7.00**)	**6.36** (**5.35**)	**0.0024**	**0.0439**
	IL-12p70	3.44 (2.32)	3.22 (1.99)	0.44	0.61
	IL-13	7.88 (16.93)	6.54 (14.78)	0.65	0.73
Monocyte activation markers (ng/mL)	CD14	2049.88 (1084.87)	2295.11 (1009.77)	0.13	0.40
	CD163	523.98 (316.92)	495.54 (235.2)	0.70	0.74
Neuroinflammatory markers (ng/mL)	NGAL	105.76 (57.97)	112.56 (59.23)	0.35	0.61
	**MMP-9**	**861.9** (**803.59**)	**1021.69** (**1264.14**)	**0.0471**	0.27
	YKL-40	19.92 (17.27)	22.68 (18.24)	0.49	0.62

Group data is presented as raw median (IQR). Statistical tests were conducted on log-scaled data. Analyses were corrected for multiple comparisons using the Benjamini-Hochberg procedure.

Supplementary Table 2. Linear mixed-effects models

This table presents results from linear mixed-effects model examining child serum marker trajectories from birth to 5 years in children who are HIV-exposed uninfected (**CHEU**) and their HIV-unexposed (**CHU**) peers. Beta coefficients (**β**), standard errors (**SE**), t-values, degrees of freedom (**DF**), p-values, and Benjamini–Hochberg adjusted p-values (**BH p-values**) are reported for each orthogonal polynomial contrast (**OPC**): baseline (6 weeks), linear, quadratic, and cubic trajectories. **N obs** indicates the number of observations, **N sub** the number of subjects, and **ICC** the intraclass correlation coefficient from the model.

CHU are the reference group; estimates in CHU sections represent serum marker trends over time in these children. CHEU rows present the difference in serum marker trajectories relative to CHU at each corresponding OPC. A significant β at baseline for CHEU ($p<0.05$) indicates significantly different serum marker levels at birth compared to CHU. Significant interaction terms indicate that the longitudinal trajectory of a certain marker over time is different between groups; differences can be linear, quadratic, or cubic based on trajectory shapes.

Marker type	Marker	Group	OPC	β	SE	T-value	DF	P-value	BH p-value	N obs	N sub	ICC
Pro-inflammatory	GM-CSF	CHU	Baseline	4.009	0.057	70.59	206.6945	**<0.001**	**0.003**	623	215	0.295
			Linear	1.022	0.076	13.39	473.2402	**<0.001**	**0.003**			
			Quadratic	-1.105	0.076	-14.49	459.1556	**<0.001**	**0.003**			
			Cubic	0.684	0.077	8.92	457.3599	**<0.001**	**0.003**			
		CHEU	Baseline	-0.259	0.093	-2.80	215.4157	**0.006**	**0.015**			
			Linear	0.015	0.127	0.12	485.4109	0.906	0.932			
			Quadratic	0.022	0.127	0.17	473.4586	0.865	0.909			
			Cubic	0.103	0.127	0.81	460.8547	0.417	0.561			
	IFNγ	CHU	Baseline	2.415	0.041	58.58	177.6853	**<0.001**	**0.003**	624	215	0.113
			Linear	0.966	0.070	13.84	476.9073	**<0.001**	**0.003**			
			Quadratic	-0.467	0.070	-6.66	460.8962	**<0.001**	**0.003**			
			Cubic	-0.276	0.071	-3.91	457.8532	**<0.001**	**0.003**			
		CHEU	Baseline	-0.035	0.068	-0.51	188.2939	0.610	0.739			
			Linear	0.227	0.116	1.96	490.0138	0.050	0.101			
			Quadratic	-0.005	0.116	-0.04	477.1394	0.966	0.973			
			Cubic	-0.124	0.117	-1.06	462.0826	0.288	0.419			
	IL-1β	CHU	Baseline	0.660	0.044	14.92	189.1803	**<0.001**	**0.003**	623	215	0.212
			Linear	0.742	0.066	11.23	469.7764	**<0.001**	**0.003**			
			Quadratic	-0.288	0.066	-4.36	455.1048	**<0.001**	**0.003**			
			Cubic	-0.060	0.067	-0.90	452.1019	0.368	0.500			
		CHEU	Baseline	-0.156	0.072	-2.16	198.4475	**0.032**	0.070			
			Linear	0.268	0.110	2.45	482.8661	**0.015**	**0.035**			
			Quadratic	-0.043	0.110	-0.39	470.2656	0.697	0.823			
			Cubic	-0.100	0.110	-0.91	455.9042	0.363	0.500			

Marker type	Marker	Group	OPC	β	SE	T-value	DF	P-value	BH p-value	N obs	N sub	ICC
	IL-2	CHU	Baseline	0.651	0.041	15.72	186.8684	**<0.001**	**0.003**	624	215	0.128
			Linear	0.530	0.069	7.72	482.0314	**<0.001**	**0.003**			
			Quadratic	-0.479	0.069	-6.94	466.7276	**<0.001**	**0.003**			
			Cubic	0.207	0.070	2.97	463.8681	**0.003**	**0.008**			
		CHEU	Baseline	-0.100	0.068	-1.47	197.5510	0.144	0.244			
			Linear	0.033	0.114	0.29	494.8908	0.773	0.870			
			Quadratic	-0.027	0.114	-0.24	482.4373	0.813	0.887			
			Cubic	-0.216	0.115	-1.88	467.9056	0.061	0.119			
	IL-5	CHU	Baseline	0.958	0.050	19.00	201.3707	**<0.001**	**0.003**	624	215	0.249
			Linear	0.600	0.072	8.36	474.6514	**<0.001**	**0.003**			
			Quadratic	-0.589	0.072	-8.19	461.6827	**<0.001**	**0.003**			
			Cubic	0.180	0.072	2.48	459.6243	**0.013**	**0.031**			
		CHEU	Baseline	-0.114	0.083	-1.38	210.8371	0.169	0.283			
			Linear	-0.439	0.119	-3.68	488.1433	**<0.001**	**0.003**			
			Quadratic	0.156	0.120	1.30	476.4356	0.193	0.312			
			Cubic	-0.088	0.120	-0.74	463.1634	0.461	0.603			
	IL-6	CHU	Baseline	1.038	0.080	12.96	197.8931	**<0.001**	**0.003**	624	215	0.202
			Linear	0.520	0.121	4.30	478.9070	**<0.001**	**0.003**			
			Quadratic	-0.591	0.121	-4.88	465.0803	**<0.001**	**0.003**			
			Cubic	-0.063	0.122	-0.52	462.7333	0.605	0.739			
		CHEU	Baseline	-0.206	0.131	-1.57	207.8671	0.118	0.207			
			Linear	-0.197	0.201	-0.98	492.1695	0.326	0.465			
			Quadratic	-0.241	0.201	-1.20	480.1668	0.232	0.355			
			Cubic	-0.147	0.202	-0.73	466.4200	0.466	0.604			
	IL-7	CHU	Baseline	2.137	0.034	62.35	183.4732	**<0.001**	**0.003**	624	215	0.116
			Linear	0.326	0.058	5.65	481.4469	**<0.001**	**0.003**			
			Quadratic	-0.403	0.058	-6.94	465.8221	**<0.001**	**0.003**			
			Cubic	0.021	0.058	0.36	462.8580	0.721	0.842			
		CHEU	Baseline	-0.005	0.056	-0.08	194.2376	0.935	0.955			
			Linear	0.018	0.096	0.19	494.2747	0.849	0.899			
			Quadratic	0.043	0.096	0.44	481.7045	0.657	0.782			
			Cubic	-0.149	0.097	-1.54	466.9879	0.125	0.214			
	IL-8	CHU	Baseline	2.589	0.074	35.17	201.9011	**<0.001**	**0.003**	624	215	0.244
			Linear	0.556	0.105	5.28	475.7419	**<0.001**	**0.003**			
			Quadratic	-0.227	0.105	-2.16	462.7308	**0.032**	0.070			
			Cubic	-0.141	0.106	-1.33	460.6519	0.184	0.301			
		CHEU	Baseline	0.026	0.120	0.22	211.4342	0.830	0.892			
			Linear	0.188	0.175	1.07	489.1732	0.284	0.417			
			Quadratic	0.216	0.175	1.23	477.4727	0.220	0.344			
			Cubic	-0.062	0.176	-0.35	464.1936	0.725	0.842			
	TNFα	CHU	Baseline	2.572	0.036	71.87	169.3344	**<0.001**	**0.003**	624	215	0.105
			Linear	-0.382	0.061	-6.24	470.9187	**<0.001**	**0.003**			
			Quadratic	0.135	0.062	2.19	454.2980	**0.029**	0.065			
			Cubic	-0.097	0.062	-1.57	451.1126	0.117	0.207			

Marker type	Marker	Group	OPC	β	SE	T-value	DF	P-value	BH p-value	N obs	N sub	ICC
		CHEU	Baseline	-0.009	0.059	-0.15	179.7699	0.882	0.920			
			Linear	0.191	0.101	1.88	484.3450	0.061	0.119			
			Quadratic	0.035	0.102	0.34	471.0527	0.731	0.842			
			Cubic	-0.063	0.103	-0.62	455.5061	0.539	0.675			
Anti-inflammatory	IL-4	CHU	Baseline	3.425	0.077	44.72	198.8740	**<0.001**	**0.003**	624	215	0.245
			Linear	0.780	0.109	7.12	473.1659	**<0.001**	**0.003**			
			Quadratic	-0.623	0.110	-5.68	460.0141	**<0.001**	**0.003**			
			Cubic	-0.078	0.110	-0.71	457.9148	0.479	0.610			
		CHEU	Baseline	-0.212	0.125	-1.69	208.3310	0.092	0.170			
			Linear	0.024	0.182	0.13	486.7626	0.893	0.925			
			Quadratic	-0.044	0.182	-0.24	474.9205	0.810	0.887			
			Cubic	-0.107	0.183	-0.58	461.4938	0.559	0.694			
	IL-10	CHU	Baseline	2.484	0.042	59.68	187.1844	**<0.001**	**0.003**	624	215	0.193
			Linear	-0.418	0.063	-6.59	471.1790	**<0.001**	**0.003**			
			Quadratic	-0.390	0.064	-6.13	456.6628	**<0.001**	**0.003**			
			Cubic	0.259	0.064	4.04	454.1742	**<0.001**	**0.003**			
		CHEU	Baseline	-0.184	0.068	-2.70	196.9789	**0.008**	**0.020**			
			Linear	-0.187	0.105	-1.77	484.9374	0.077	0.144			
			Quadratic	-0.032	0.106	-0.30	472.3858	0.763	0.865			
			Cubic	-0.080	0.106	-0.75	458.0261	0.451	0.596			
	IL-12p70	CHU	Baseline	1.152	0.040	28.88	184.2989	**<0.001**	**0.003**	624	215	0.180
			Linear	0.262	0.062	4.24	470.8358	**<0.001**	**0.003**			
			Quadratic	-0.421	0.062	-6.79	455.9783	**<0.001**	**0.003**			
			Cubic	0.210	0.062	3.35	453.3863	**<0.001**	**0.003**			
		CHEU	Baseline	-0.072	0.065	-1.10	194.1808	0.272	0.408			
			Linear	0.203	0.103	1.97	484.5984	**0.049**	0.101			
			Quadratic	-0.066	0.103	-0.64	471.8905	0.525	0.663			
			Cubic	-0.164	0.103	-1.59	457.3127	0.113	0.206			
	IL-13	CHU	Baseline	1.919	0.089	21.55	204.4346	**<0.001**	**0.003**	624	215	0.319
			Linear	0.742	0.116	6.40	467.3485	**<0.001**	**0.003**			
			Quadratic	-0.920	0.116	-7.93	455.6742	**<0.001**	**0.003**			
			Cubic	0.235	0.117	2.01	454.0130	**0.045**	0.095			
		CHEU	Baseline	-0.132	0.145	-0.91	213.1386	0.366	0.500			
			Linear	-0.059	0.193	-0.31	480.9932	0.758	0.865			
			Quadratic	0.140	0.193	0.72	469.8473	0.470	0.604			
			Cubic	-0.247	0.193	-1.28	457.3701	0.202	0.323			
Monocyte activation	CD14	CHU	Baseline	7.584	0.020	370.91	191.0327	**<0.001**	**0.003**	625	215	0.237
			Linear	0.219	0.030	7.43	468.0744	**<0.001**	**0.003**			
			Quadratic	-0.197	0.030	-6.66	454.3850	**<0.001**	**0.003**			
			Cubic	0.047	0.030	1.57	452.1787	0.117	0.207			
		CHEU	Baseline	0.086	0.033	2.58	200.0964	**0.011**	**0.026**			
			Linear	-0.012	0.049	-0.24	480.8668	0.810	0.887			
			Quadratic	0.045	0.049	0.92	468.6972	0.361	0.500			
			Cubic	-0.125	0.049	-2.54	457.2720	**0.011**	**0.026**			

Marker type	Marker	Group	OPC	β	SE	T-value	DF	P-value	BH p-value	N obs	N sub	ICC
	CD163	CHU	Baseline	6.403	0.031	203.42	208.1133	**<0.001**	**0.003**	625	215	0.402
			Linear	-0.068	0.037	-1.84	460.4099	0.067	0.129			
			Quadratic	-0.162	0.037	-4.40	450.3383	**<0.001**	**0.003**			
			Cubic	-0.029	0.037	-0.79	449.1004	0.430	0.573			
		CHEU	Baseline	-0.013	0.051	-0.26	215.5244	0.796	0.887			
			Linear	-0.095	0.061	-1.55	472.5353	0.121	0.210			
			Quadratic	-0.004	0.061	-0.06	462.5814	0.949	0.962			
			Cubic	0.120	0.061	1.96	453.2366	0.050	0.101			
Neuroinflammatory	NGAL	CHU	Baseline	4.787	0.029	166.89	188.4458	**<0.001**	**0.003**	624	215	0.156
			Linear	0.020	0.046	0.45	478.2307	0.655	0.782			
			Quadratic	-0.317	0.046	-6.88	463.3064	**<0.001**	**0.003**			
			Cubic	0.262	0.046	5.65	460.6166	**<0.001**	**0.003**			
		CHEU	Baseline	-0.064	0.047	-1.36	199.4345	0.176	0.291			
			Linear	0.146	0.076	1.91	492.0003	0.056	0.112			
			Quadratic	0.018	0.076	0.23	479.0493	0.819	0.887			
			Cubic	0.080	0.077	1.04	466.0983	0.300	0.432			
	MMP-9	CHU	Baseline	6.529	0.042	154.93	200.0863	**<0.001**	**0.003**	624	215	0.209
			Linear	0.502	0.063	7.97	479.3032	**<0.001**	**0.003**			
			Quadratic	-0.346	0.063	-5.48	465.6838	**<0.001**	**0.003**			
			Cubic	0.032	0.064	0.51	463.3946	0.611	0.739			
		CHEU	Baseline	0.083	0.069	1.21	210.4898	0.228	0.353			
			Linear	0.186	0.105	1.77	492.8931	0.077	0.144			
			Quadratic	0.021	0.105	0.20	480.6255	0.843	0.899			
			Cubic	-0.003	0.105	-0.03	468.4308	0.977	0.977			
	YKL-40	CHU	Baseline	3.297	0.039	83.48	190.6215	**<0.001**	**0.003**	625	215	0.396
			Linear	-0.233	0.047	-5.01	444.9912	**<0.001**	**0.003**			
			Quadratic	-0.057	0.047	-1.23	434.2399	0.218	0.344			
			Cubic	-0.094	0.047	-2.01	432.9085	**0.045**	0.095			
		CHEU	Baseline	0.073	0.064	1.13	197.7634	0.258	0.391			
			Linear	0.071	0.078	0.92	457.8959	0.358	0.500			
			Quadratic	-0.085	0.078	-1.09	447.2502	0.275	0.408			
			Cubic	0.183	0.077	2.37	437.2982	**0.018**	**0.041**			

Supplementary Table 3. Group differences in parietal brain metabolite levels at 6–7 years

Brain region	Neurometabolite	MRS measurement	CHU	CHEU	β	95% CI	P-value
**Midline parietal grey matter**	**Glutamate**	**Ratios to creatine**	**1.68** (**0.23**)	**1.60** (**0.27**)	**0.10**	(**0.03 to 0.63**)	**0.0308**
		Absolute concentrations	11.98 (2.52)	11.69 (2.55)	0.07	(-0.08 to 0.55)	0.14
	Myo-inositol	Ratios to creatine	0.85 (0.07)	0.84 (0.06)	0.06	(-0.08 to 0.50)	0.16
		Absolute concentrations	6.15 (1.22)	6.02 (0.71)	0.04	(-0.15 to 0.43)	0.35
	N-acetyl-aspartate	Ratios to creatine	1.38 (0.12)	1.36 (0.1)	0.02	(-0.24 to 0.37)	0.68
		Absolute concentrations	10.15 (2.1)	9.69 (1.31)	0.02	(-0.23 to 0.38)	0.64
	Total choline	Ratios to creatine	0.17 (0.02)	0.17 (0.02)	-0.01	(-0.37 to 0.32)	0.90
		Absolute concentrations	1.24 (0.24)	1.25 (0.25)	-0.02	(-0.42 to 0.27)	0.68
	Total creatine	Absolute concentrations	7.34 (1.15)	7.06 (1.00)	0.00	(-0.33 to 0.33)	0.99
**Left parietal white matter**	Glutamate	Ratios to creatine	1.41 (0.28)	1.42 (0.3)	0.03	(-0.28 to 0.49)	0.58
		Absolute concentrations	10.03 (1.72)	10.62 (3.15)	-0.06	(-1.07 to 0.39)	0.34
	Myo-inositol	Ratios to creatine	0.86 (0.11)	0.87 (0.08)	-0.04	(-0.45 to 0.17)	0.39
		Absolute concentrations	6.12 (1.07)	6.4 (1.04)	-0.10	(-1.11 to 0.04)	0.0646
	N-acetyl-aspartate	Ratios to creatine	1.55 (0.15)	1.52 (0.19)	0.06	(-0.16 to 0.61)	0.24
		Absolute concentrations	11.1 (1.84)	11.31 (2.78)	0.01	(-0.66 to 0.75)	0.90
	**Total choline**	**Ratios to creatine**	**0.25** (**0.03**)	**0.23** (**0.04**)	**0.13**	(**0.04 to 0.82**)	**0.0295**
		Absolute concentrations	1.72 (0.28)	1.66 (0.34)	0.00	(-0.72 to 0.67)	0.94
	Total creatine	Absolute concentrations	7.01 (1.06)	7.18 (0.81)	-0.03	(-0.80 to 0.42)	0.53

Group data is presented as raw median (IQR); statistical tests were conducted on scaled data. High-quality MRS data available in the midline parietal grey matter: n=131 CHU and n=57 CHEU (ratios to creatine); n=127 CHU and n=57 CHEU (absolute concentrations, in millimolar). In the left parietal white

matter: n=116 CHU and n=47 CHEU (ratios to creatine); n=74 CHU and n=16 CHEU (absolute concentrations, in millimolar). **CHEU**: Children who are HIV-Exposed and Uninfected; **CHU**: Children who are HIV-Unexposed; **MRS**: Magnetic Resonance Spectroscopy; **β**: Effect size.

Supplementary Table 4. Associations between maternal/child serum marker levels at different timepoints and child neurometabolite ratios to creatine at 6–7 years

A full Statistical Analysis Report, including all unadjusted and adjusted results, has been deposited on OSF and is publicly available under DOI: 10.17605/OSF.IO/7N94C.

Maternal serum marker levels during pregnancy
Glutamate: Maternal **IL-8** levels during pregnancy were negatively associated with glutamate ratios in the left parietal white matter of children who are HEU (β=−0.73, 95% CI −1.13 to −0.33, p=0.0004). No significant associations were observed in HU peers.
Total choline: In the midline parietal grey matter, maternal **IL-6** and **IL-4** were positively associated with total choline ratios in HU children (IL-6: β=0.25, 95% CI 0.10 to 0.40, p=0.0017; IL-4: β=0.21, 95% CI 0.06 to 0.37, p=0.0077). Maternal HIV significantly altered these relationships, and no significant associations were detected in children who are HEU.
N-acetyl-aspartate: Maternal **IL-8** was negatively associated with lower N-acetyl-aspartate ratios in the left parietal white matter of HEU children only (β=−0.69, 95% CI −1.22 to −0.17, p=0.0101).

Child serum marker trajectories from 6 weeks to 5 years
Myo-inositol: Slopes of **IL-8**, **TNFα**, and **IL-4** from infancy to 5 years were negatively associated with myo-inositol ratios in the midline parietal grey matter of HU children (IL-8: β=−0.28, 95% CI −0.49 to −0.06, p=0.0124; TNFα: β=−0.44, 95% CI −0.72 to −0.16, p=0.0023; IL-4: β=−0.29, 95% CI −0.49 to −0.09, p=0.0048). No significant associations were observed in HEU peers.
Total choline: In children who are HEU, **IL-8** trajectories were negatively associated with total choline ratios in the midline parietal grey matter (β=−0.34, 95% CI −0.68 to −0.01, p=0.0461). In HU children, TNFα trajectories were negatively associated with total choline ratios in the left parietal white matter (β=−0.32, 95% CI −0.54 to −0.09, p=0.0060).

Week 6 serum marker levels

Myo-inositol: **sCD14** levels during infancy were negatively associated with lower myo-inositol ratios in the midline parietal grey matter of HU children (β=−1.43, 95% CI −2.53 to −0.34, p=0.0107). No significant associations were observed in HEU peers.

Year 2 serum marker levels

Glutamate: In HU children, **IL-13** was positively associated with left parietal white matter glutamate ratios (β=0.28, 95% CI 0.08 to 0.47, p=0.0058). No significant associations were observed in HEU peers.

Year 3 serum marker levels

Glutamate: At age 3 years, **IL-4** and **IL-13** were negatively associated with glutamate ratios in the left parietal white matter of children who are HEU (IL-4: β=−0.87, 95% CI −1.36 to −0.37, p=0.0007; IL-13: β=−0.67, 95% CI −1.06 to −0.28, p=0.0009). No significant associations were observed in HU peers.

Year 5 serum marker levels

Glutamate: At age 5 years, **IL-8** was negatively associated with glutamate ratios in the midline parietal grey matter of HU children (β=−0.21, 95% CI −0.36 to −0.05, p=0.0100). No significant associations were observed in HEU peers.

Myo-inositol: Several markers measured at age 5 years were negatively associated with myo-inositol ratios in HU children: **IL-8** (β=−0.19, 95% CI −0.35 to −0.04, p=0.0143), **TNFα** (β=−0.44, 95% CI −0.71 to −0.16, p=0.0022), **IL-4** (β=−0.22, 95% CI −0.40 to −0.04, p=0.0192), **IL-13** (β=−0.20, 95% CI −0.35 to −0.04, p=0.0164), and **NGAL** (β=−0.66, 95% CI −1.25 to −0.07, p=0.0298). Only **IL-4** showed a significant association in children who are HEU (β=0.22, 95% CI 0.01 to 0.44, p=0.0399).

Total choline: In HEU children, **IL-1β** was negatively associated with total choline ratios in the midline parietal grey matter (β=−0.63, 95% CI −1.16 to −0.09, p=0.0217). No significant associations were observed in HU peers.

Supplementary Table 5. Adjusted linear models exploring associations between maternal/child serum marker levels at different timepoints and midline parietal grey matter neurometabolite absolute concentrations at 6–7 years

							CHU		CHEU	
**Serum**	**Timepoint**	**n CHU**	**n CHEU**	**Neurometabolite**	**Marker type**	**Marker**	**β** (95% CI)	**P-value**	**β** (95% CI)	**P-value**
Child	6 weeks	70	40	Choline	Anti-inflammatory	IL-4	–	–	–0.27 (–0.51 to –0.03)	**0.0310**
	2 years	76	39	Glutamate	Monocyte activation	CD14	–0.86 (–1.54 to –0.18)	**0.0135**	–	–
				Myo-inositol	Pro-inflammatory	TNFα	–	–	–0.47 (–0.92 to –0.02)	**0.0422**
					Monocyte activation	CD14	–0.74 (–1.43 to –0.06)	**0.0333**	–	–
	3 years	69	38	Glutamate	Pro-inflammatory	IFNγ	–0.38 (–0.71 to –0.04)	**0.0288**	–	–
					Monocyte activation	CD14	–0.71 (–1.41 to –0.01)	**0.0495**	–	–
				N-acetyl-aspartate	Pro-inflammatory	IL-5	0.28 (0.03 to 0.54)	**0.0321**	–	–

Linear regression models with robust standard errors. Models were adjusted for child age, child sex, and voxel tissue composition. **CHEU**: Children who are HIV-Exposed and Uninfected; **CHU**: Children who are HIV-Unexposed; **β**: Effect size.

Please note: Association analyses using child absolute neurometabolite concentrations in the left parietal white matter are not presented because the small sample size in this region (CHEU n=16) precluded adequately adjusted models and yielded unstable unadjusted estimates. As prespecified, only adjusted, BH-corrected associations are reported.

Supplementary Table 6. Sensitivity analyses

6.1. Maternal age at delivery

								CHU		CHEU	
**Serum**	**Timepoint**	**Brain region**	**n CHU**	**n CHEU**	**Neurometabolite**	**Marker type**	**Marker**	**β** (95% CI)	**P-value**	**β** (95% CI)	**P-value**
Maternal	Pregnancy	GM	99	54	Choline	Pro-inflammatory	IL-6	0.25 (0.10 to 0.41)	**0.0017**	–	–
						Anti-inflammatory	IL-4	0.21 (0.06 to 0.37)	**0.0070**	–	–
		WM	88	44	Glutamate	Pro-inflammatory	IL-8	–	–	–0.75 (–1.13 to –0.36)	**0.0002**
					N-acetyl-aspartate	Pro-inflammatory	IL-8	–	–	–0.69 (–1.22 to –0.15)	**0.0131**
Child	6 weeks	GM	75	40	Myo-inositol	Monocyte activation	CD14	–1.37 (–2.44 to –0.29)	**0.0135**	–	–
	2 years	WM	72	32	Glutamate	Anti-inflammatory	IL-13	0.26 (0.06 to 0.46)	**0.0058**	–	–
	3 years	WM	62	31	Glutamate	Anti-inflammatory	IL-4	–	–	–0.87 (–1.36 to –0.37)	**0.0008**
							IL-13	–	–	–0.67 (–1.07 to –0.28)	**0.0010**
	5 years	GM	81	45	Glutamate	Pro-inflammatory	IL-8	–0.21 (–0.37 to –0.05)	**0.0116**	–	–
					Myo-inositol	Pro-inflammatory	IL-8	–0.20 (–0.35 to –0.05)	**0.0095**	–	–
							TNFα	–0.48 (–0.76 to –0.20)	**0.0008**	–	–
						Anti-inflammatory	IL-4	–0.24 (–0.42 to –0.06)	**0.0098**	0.25 (0.03 to 0.46)	**0.0235**
							IL-13	–0.22 (–0.38 to –0.06)	**0.0072**	–	–
						Neuroinflammatory	NGAL	–0.69 (–1.28 to –0.10)	**0.0222**	–	–
					Choline	Pro-inflammatory	IL-1β	–	–	–0.68 (–1.21 to –0.14)	**0.0134**

Linear regression models with robust standard errors. Adjusted for child age, child sex, voxel composition, and **maternal age at delivery**.

6.2. Maternal alcohol use during pregnancy

								CHU		CHEU	
**Serum**	**Timepoint**	**Brain region**	**n CHU**	**n CHEU**	**Neurometabolite**	**Marker type**	**Marker**	**β** (95% CI)	**P-value**	**β** (95% CI)	**P-value**
Maternal	Pregnancy	GM	99	54	Choline	Pro-inflammatory	IL-6	0.25 (0.09 to 0.40)	**0.0020**	–	–
						Anti-inflammatory	IL-4	0.21 (0.05 to 0.36)	**0.0092**	–	–
		WM	88	44	Glutamate	Pro-inflammatory	IL-8	–	–	–0.76 (–1.20 to –0.31)	**0.0010**
					N-acetyl-aspartate	Pro-inflammatory	IL-8	–	–	–0.86 (–1.31 to –0.41)	**0.0002**
Child	6 weeks	GM	75	40	Myo-inositol	Monocyte activation	CD14	–1.44 (–2.54 to –0.34)	**0.0105**	–	–
	2 years	WM	72	32	Glutamate	Anti-inflammatory	IL-13	0.29 (0.07 to 0.50)	**0.0096**	–	–
	3 years	WM	62	31	Glutamate	Anti-inflammatory	IL-4	–	–	–0.84 (–1.40 to –0.27)	**0.0043**
							IL-13	–	–	–0.65 (–1.10 to –0.21)	**0.0048**
	5 years	GM	81	45	Glutamate	Pro-inflammatory	IL-8	–0.19 (–0.34 to –0.04)	**0.0139**	–	–
					Myo-inositol	Pro-inflammatory	IL-8	–0.18 (–0.33 to –0.03)	**0.0211**	–	–
							TNFα	–0.41 (–0.68 to –0.14)	**0.0036**	–	–
						Anti-inflammatory	IL-4	–0.21 (–0.38 to –0.04)	**0.0178**	0.18 (–0.03 to 0.40)	0.09
							IL-13	–0.20 (–0.35 to –0.05)	**0.0115**	–	–
						Neuroinflammatory	NGAL	–0.62 (–1.23 to –0.01)	**0.0449**	–	–
					Choline	Pro-inflammatory	IL-1β	–	–	–0.79 (–1.36 to –0.22)	**0.0067**

Linear regression models with robust standard errors. Adjusted for child age, child sex, voxel composition, and **maternal alcohol use during pregnancy**.

6.3. Maternal depression during pregnancy

								CHU		CHEU	
**Serum**	**Timepoint**	**Brain region**	**n CHU**	**n CHEU**	**Neurometabolite**	**Marker type**	**Marker**	**β** (95% CI)	**P-value**	**β** (95% CI)	**P-value**
Maternal	Pregnancy	GM	99	54	Choline	Pro-inflammatory	IL-6	0.21 (0.07 to 0.36)	**0.0052**	–	–
						Anti-inflammatory	IL-4	0.18 (0.02 to 0.33)	**0.0284**	–	–
		WM	88	44	Glutamate	Pro-inflammatory	IL-8	–	–	–0.74 (–1.15 to –0.33)	**0.0005**
					N-acetyl-aspartate	Pro-inflammatory	IL-8	–	–	–0.70 (–1.23 to –0.17)	**0.0100**
Child	6 weeks	GM	75	40	Myo-inositol	Monocyte activation	CD14	–1.41 (–2.50 to –0.33)	**0.0112**	–	–
	2 years	WM	72	32	Glutamate	Anti-inflammatory	IL-13	0.28 (0.08 to 0.47)	**0.0064**	–	–
	3 years	WM	62	31	Glutamate	Anti-inflammatory	IL-4	–	–	–0.86 (–1.36 to –0.36)	**0.0010**
							IL-13	–	–	–0.67 (–1.08 to –0.27)	**0.0013**
	5 years	GM	81	45	Glutamate	Pro-inflammatory	IL-8	–0.20 (–0.38 to –0.03)	**0.0228**	–	–
					Myo-inositol	Pro-inflammatory	IL-8	–0.22 (–0.39 to –0.05)	**0.0121**	–	–
							TNFα	–0.45 (–0.73 to –0.16)	**0.0023**	–	–
						Anti-inflammatory	IL-4	–0.24 (–0.42 to –0.06)	**0.0114**	0.23 (0.02 to 0.45)	**0.0349**
							IL-13	–0.22 (–0.39 to –0.05)	**0.0113**	–	–
						Neuroinflammatory	NGAL	–0.69 (–1.34 to –0.05)	**0.0362**	–	–
					Choline	Pro-inflammatory	IL-1β	–	–	–0.65 (–1.19 to –0.11)	**0.0184**

Linear regression models with robust standard errors. Adjusted for child age, child sex, voxel composition, and **maternal depression during pregnancy**.

6.4. Maternal smoking during pregnancy

								CHU		CHEU	
**Serum**	**Timepoint**	**Brain region**	**n CHU**	**n CHEU**	**Neurometabolite**	**Marker type**	**Marker**	**β** (95% CI)	**P-value**	**β** (95% CI)	**P-value**
Maternal	Pregnancy	GM	99	54	Choline	Pro-inflammatory	IL-6	0.23 (0.08 to 0.38)	**0.0030**	–	–
						Anti-inflammatory	IL-4	0.21 (0.07 to 0.35)	**0.0041**	–	–
		WM	88	44	Glutamate	Pro-inflammatory	IL-8	–	–	–0.74 (–1.16 to –0.33)	**0.0005**
					N-acetyl-aspartate	Pro-inflammatory	IL-8	–	–	–0.70 (–1.22 to –0.18)	**0.0092**
Child	6 weeks	GM	75	40	Myo-inositol	Monocyte activation	CD14	–1.52 (–2.59 to –0.45)	**0.0059**	–	–
	2 years	WM	72	32	Glutamate	Anti-inflammatory	IL-13	0.27 (0.09 to 0.46)	**0.0045**	–	–
	3 years	WM	62	31	Glutamate	Anti-inflammatory	IL-4	–	–	–0.89 (–1.38 to –0.40)	**0.0006**
							IL-13	–	–	–0.69 (–1.07 to –0.31)	**0.0006**
	5 years	GM	81	45	Glutamate	Pro-inflammatory	IL-8	–0.21 (–0.36 to –0.05)	**0.0117**	–	–
					Myo-inositol	Pro-inflammatory	IL-8	–0.19 (–0.35 to –0.04)	**0.0161**	–	–
							TNFα	–0.44 (–0.72 to –0.16)	**0.0025**	–	–
						Anti-inflammatory	IL-4	–0.22 (–0.40 to –0.04)	**0.0161**	0.23 (0.01 to 0.45)	**0.0398**
							IL-13	–0.20 (–0.36 to –0.04)	**0.0146**	–	–
						Neuroinflammatory	NGAL	–0.68 (–1.31 to –0.05)	**0.0351**	–	–
					Choline	Pro-inflammatory	IL-1β	–	–	–0.61 (–1.15 to –0.07)	**0.0268**

Linear regression models with robust standard errors. Adjusted for child age, child sex, voxel composition, and **maternal smoking during pregnancy**.

6.5. Exclusive breastfeeding duration

								CHU		CHEU	
**Serum**	**Timepoint**	**Brain region**	**n CHU**	**n CHEU**	**Neurometabolite**	**Marker type**	**Marker**	**β** (95% CI)	**P-value**	**β** (95% CI)	**P-value**
Maternal	Pregnancy	GM	99	54	Choline	Pro-inflammatory	IL-6	0.25 (0.10 to 0.41)	**0.0015**	–	–
						Anti-inflammatory	IL-4	0.22 (0.06 to 0.38)	**0.0068**	–	–
		WM	88	44	Glutamate	Pro-inflammatory	IL-8	–	–	–0.74 (–1.15 to –0.32)	**0.0006**
					N-acetyl-aspartate	Pro-inflammatory	IL-8	–	–	–0.69 (–1.21 to –0.17)	**0.0097**
Child	6 weeks	GM	75	40	Myo-inositol	Monocyte activation	CD14	–1.46 (–2.61 to –0.31)	**0.0137**	–	–
	2 years	WM	72	32	Glutamate	Anti-inflammatory	IL-13	0.28 (0.08 to 0.47)	**0.0061**	–	–
	3 years	WM	62	31	Glutamate	Anti-inflammatory	IL-4	–	–	–0.88 (–1.42 to –0.34)	**0.0016**
							IL-13	–	–	–0.71 (–1.16 to –0.25)	**0.0026**
	5 years	GM	81	45	Glutamate	Pro-inflammatory	IL-8	–0.21 (–0.37 to –0.06)	**0.0069**	–	–
					Myo-inositol	Pro-inflammatory	IL-8	–0.19 (–0.35 to –0.04)	**0.0150**	–	–
							TNFα	–0.44 (–0.71 to –0.16)	**0.0023**	–	–
						Anti-inflammatory	IL-4	–0.22 (–0.40 to –0.04)	**0.0194**	0.22 (0.01 to 0.44)	**0.0387**
							IL-13	–0.20 (–0.36 to –0.04)	**0.0168**	–	–
						Neuroinflammatory	NGAL	–0.67 (–1.26 to –0.08)	**0.0275**	–	–
					Choline	Pro-inflammatory	IL-1β	–	–	–0.65 (–1.16 to –0.14)	**0.0134**

Linear regression models with robust standard errors. Adjusted for child age, child sex, voxel composition, and **exclusive breastfeeding duration**.

Supplementary Table 7. Early Learning Outcome Measure scores at 6–7 years

**Developmental domain**	**CHU** (n=116)	**CHEU** (n=56)	**β**	**95% CI**	**P-value**
Cognitive development	10.7 (5.7)	10.7 (5.0)	0.50	(-0.57 to 1.93)	0.39
Fine motor coordination and visual motor integration	17.8 (2.4)	17.9 (3.2)	0.00	(-0.85 to 0.24)	0.57
Gross motor development	11.5 (6.7)	12.9 (7.8)	-0.03	(-1.63 to 1.47)	0.81
Emergent mathematics	13.6 (4.5)	14.6 (7.1)	0.00	(-1.34 to 1.24)	0.88
**Emergent literacy and language**	**13.6** (**6.2**)	**11.2** (**5.3**)	**1.95**	(**0.53 to 3.37**)	**0.0084**
Overall school readiness score	68.1 (15.5)	65.7 (17.2)	1.94	(-1.92 to 5.93)	0.33

Wilcoxon Rank Sum (Mann Whitney U) tests. Group data is median (IQR). **CHEU**: Children who are HIV-Exposed and Uninfected; **CHU**: Children who are HIV-Unexposed; **β**: Effect size. More information on the South African developmental tool *Early Learning Outcome Measures* can be found at: https://datadrive2030.co.za/elom-origins/

Supplementary Figures

Supplementary Figure 1. Maternal serum marker levels during pregnancy

Raw data plotted. Statistical tests were conducted on log-scaled data.

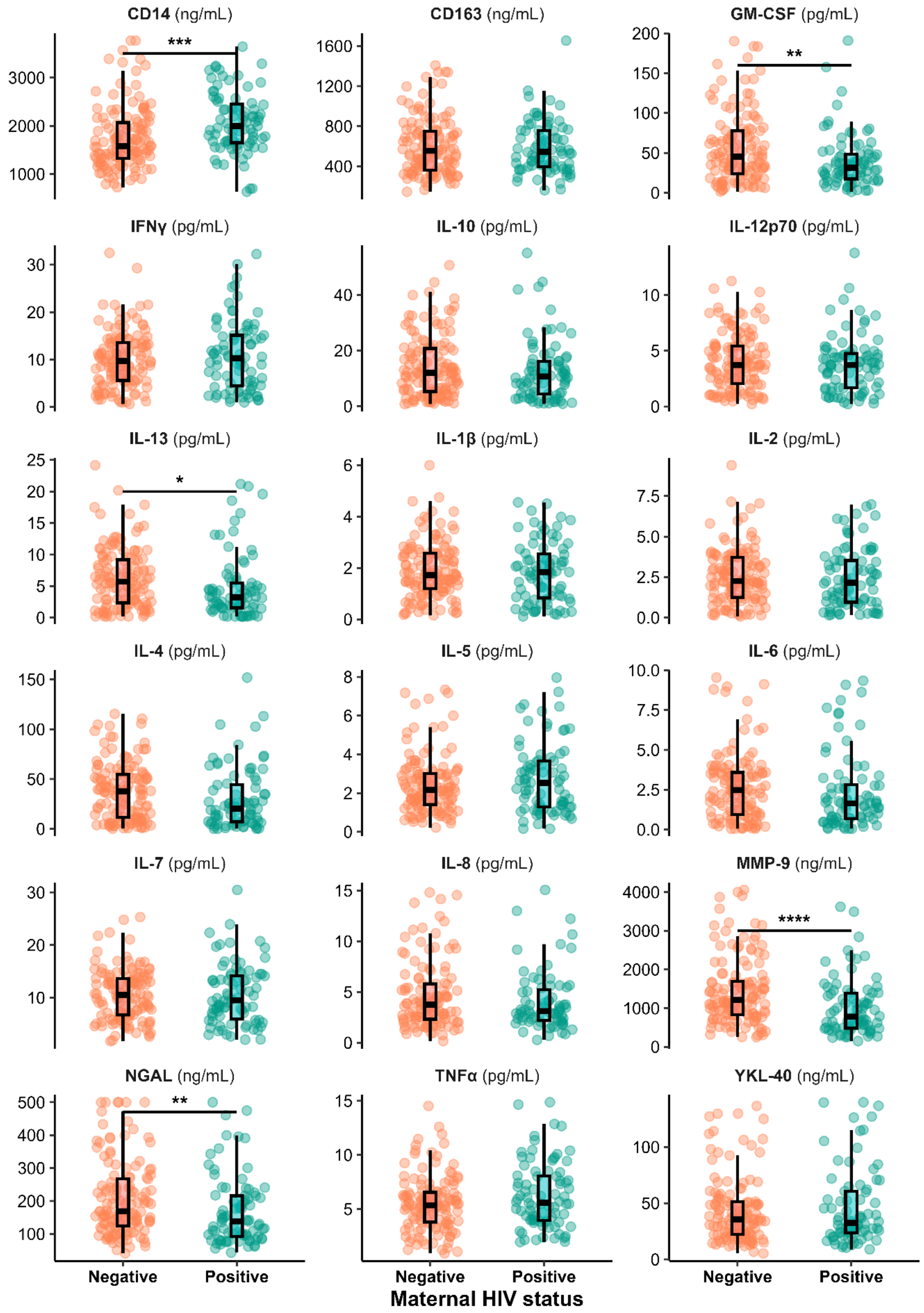

Supplementary Figure 2. Child neurometabolite absolute concentrations at 6–7 years of age

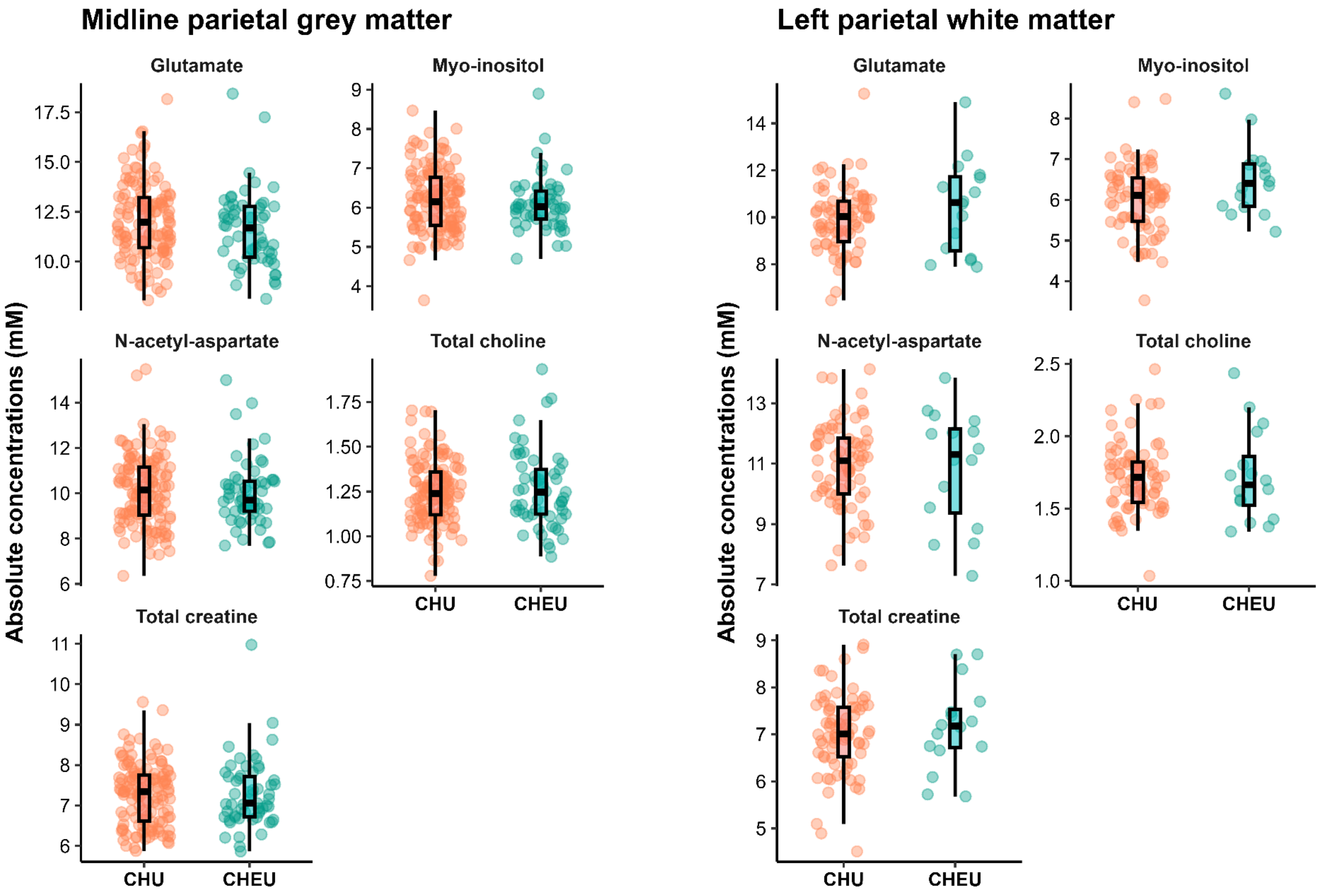

Mediation analyses

To investigate whether maternal/child serum immune markers and/or child neurometabolite ratios mediated the relationship between maternal HIV status and child neurodevelopmental outcomes, three sets of mediation models were conducted using structural equation modeling (SEM) within the *lavaan* package in R. All models were fitted using full information maximum likelihood (FIML) to account for missing data and non-parametric bootstrapping with 5,000 resamples to obtain robust standard errors and percentile-based 95% confidence intervals for indirect effects.

Model specifications were guided by prior biological hypotheses and upstream analyses that identified significant associations between maternal HIV status, maternal/child serum markers, child neurometabolite ratios, and Early Learning Outcomes Measure (ELOM) scores at 6–7 years of age. Separate models were run for each candidate serum marker or marker slope, neurometabolite ratio, or marker–neurometabolite pair.

Model 1

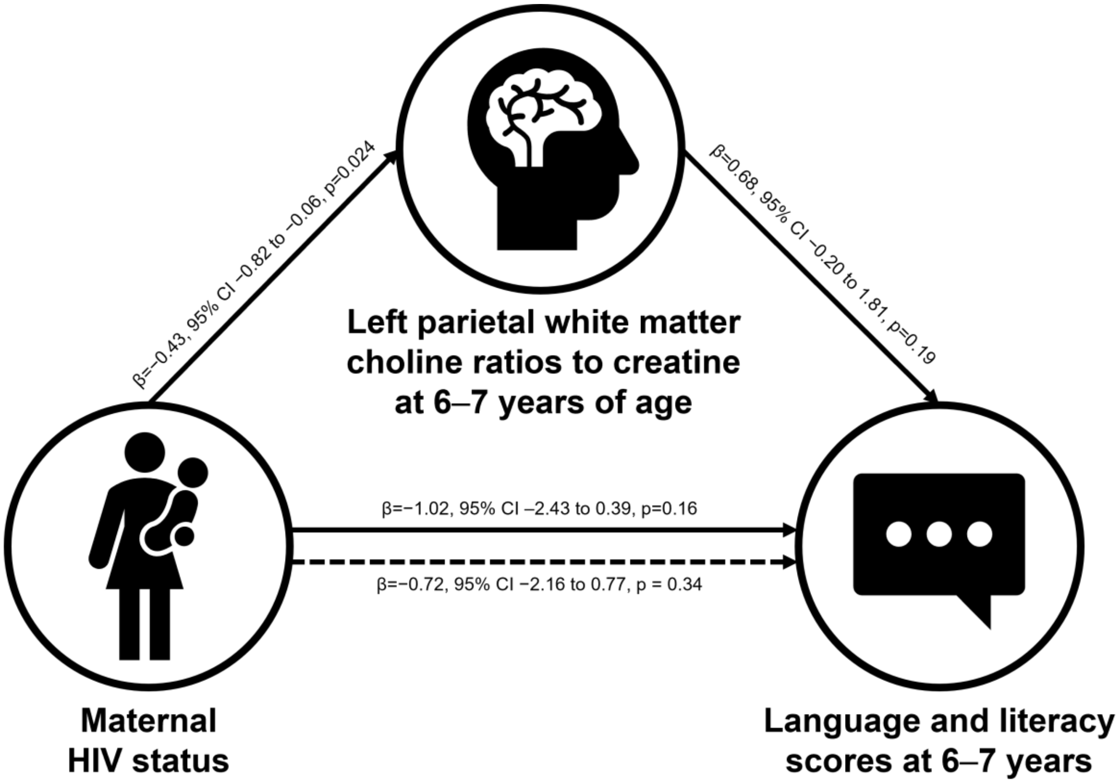

The first model evaluated whether left parietal white matter choline ratios to creatine at 6–7 years mediated the association between maternal HIV status and child ELOM language and literacy scores. In group comparisons, both choline ratios (**Supplementary Table 1**) and ELOM language and literacy scores (**Supplementary Table 6**) were significantly lower in children who are HEU compared to HU peers. Within the HU group, higher left parietal white matter choline ratios were positively associated with better language and literacy performance. These patterns provided the biological and statistical rationale for testing whether differences in choline levels could explain, at least in part, the relationship between maternal HIV exposure and child neurodevelopmental outcomes.

In this model, maternal HIV was significantly associated with lower choline ratios (β=−0.43, 95% CI −0.82 to −0.06, p=0.024). Higher choline ratios were positively associated with ELOM language and literacy scores (β=0.68, 95% CI −0.20 to 1.81, p=0.19), although this association was not statistically significant. The indirect effect of maternal HIV on ELOM scores through choline was negative but non-significant (β=−0.30, 95% CI −0.91 to 0.12, p=0.26). The total effect of maternal HIV on language and literacy scores was β=−1.02, 95% CI –2.43 to 0.39, p=0.16, and the direct effect, after accounting for choline, was β=−0.72, 95% CI −2.16 to 0.77, p = 0.34.

Model 2

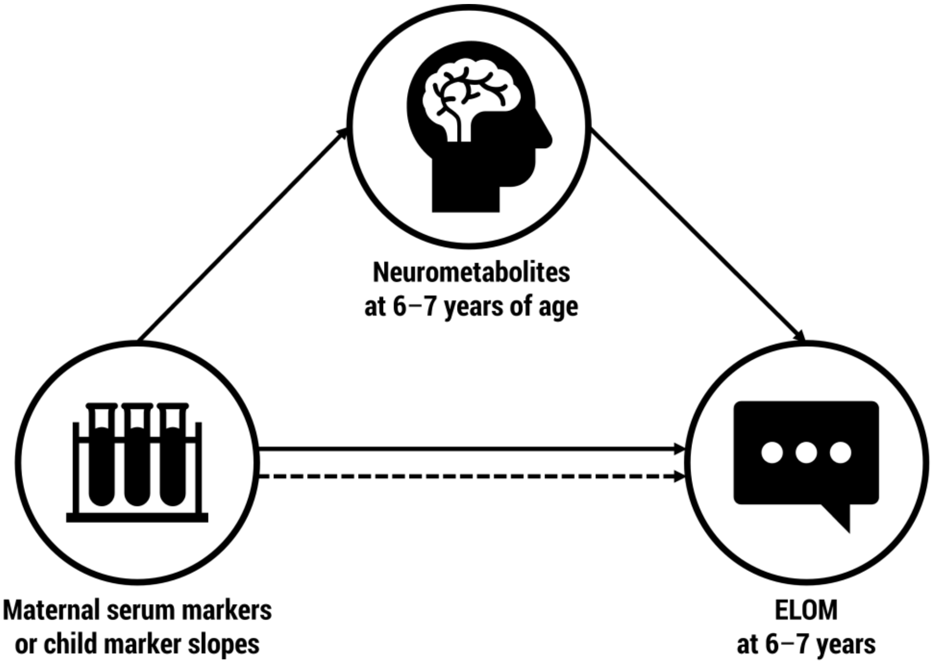

The second set of analyses extended this framework to systematically evaluate whether selected serum markers, neurometabolite ratios in either midline parietal grey matter or left parietal white matter, or marker–metabolite pairs acted as mediators of the maternal HIV → child ELOM pathway.

For each model, a different candidate mediator was explored based on upstream associations (**Table 2** and **Table 3** in main manuscript). The model structure remained the same as in Model 1, with maternal HIV as the predictor and child ELOM scores as the outcome. Each mediation model was run independently, and none reached our threshold for statistical significance.

Model 3

The third set of analyses tested serial multiple mediation to capture a potential biological cascade where maternal or child serum markers influence child neurometabolite levels, which in turn affect neurodevelopment and school readiness.

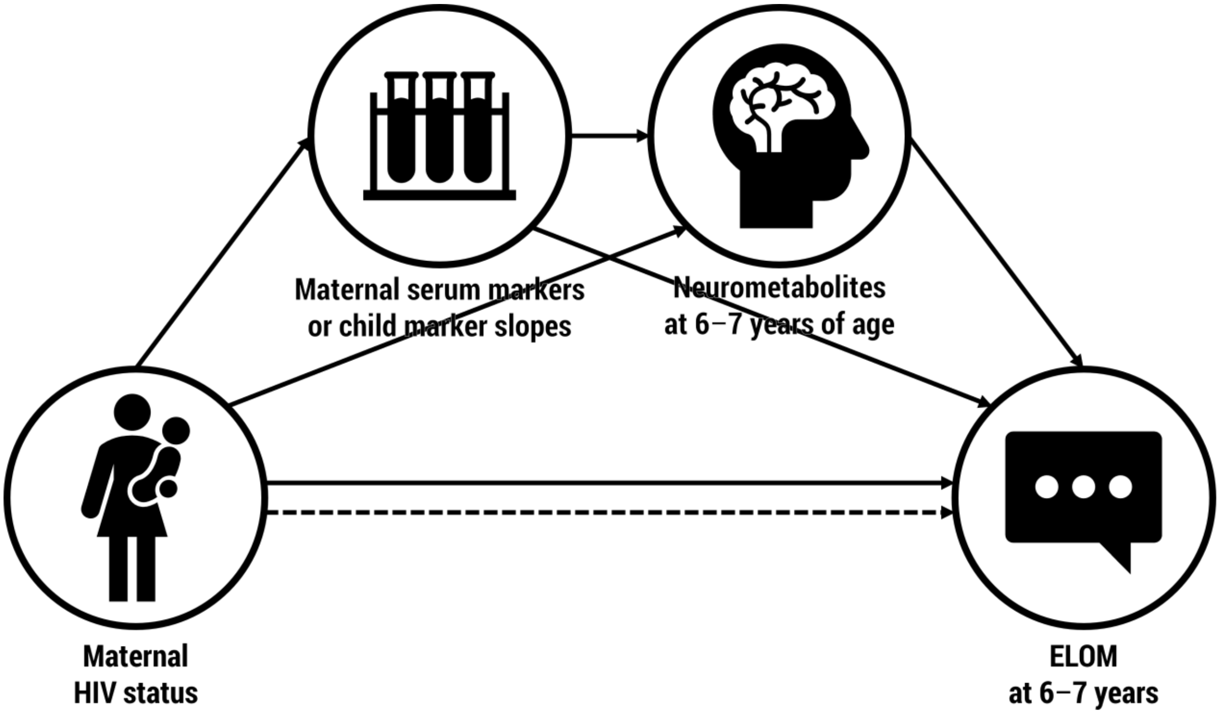

This model included two mediators in sequence:

- Predictor (X): Maternal HIV status
- Mediator 1 (M1): Serum marker (maternal or child, cross-sectional or longitudinal)

- Mediator 2 (M2): Child neurometabolite ratios to creatine (parietal grey or white matter)
- Outcome (Y): ELOM score (overall or domain-specific)

The indirect effects were decomposed into three components:

- X → M1 → Y
- X → M2 → Y
- X → M1 → M2 → Y (serial pathway)

The total indirect effect was calculated as the sum of these three pathways. Parameter estimates, standardized coefficients, and bootstrap confidence intervals were extracted to determine whether any of the indirect effects were statistically significant. Model fit indices were examined to ensure adequate model specification.

Different combinations of candidate mediators and outcomes were explored based on upstream associations. For example, IL-1β trajectories were significantly different between HEU and HU children (**Supplementary Table 3**). At age 5 years, child IL-1β concentrations were negatively associated with left parietal white matter choline ratios in the HEU group (**Table 2** in main manuscript). These choline ratios were lower in HEU compared to HU children in group comparisons (**Supplementary Table 1**), and within the HU group, higher choline ratios were positively associated with ELOM language scores. Language scores were lower in HEU versus HU children (**Supplementary Table 6**). Together, these patterns suggested a biologically plausible serial pathway in which maternal HIV status alters child early-life IL-1β trajectories, which in turn impact mid-childhood left parietal white matter choline ratios and, ultimately, language outcomes at school entry.

Each mediation model was run independently, and none reached our threshold for statistical significance.
